# Prolonged Hyperoxia Exposure is an Independent Predictor for Moderate to Severe Neurodevelopmental Impairment in Extremely Premature Neonates

**DOI:** 10.21203/rs.3.rs-7428681/v1

**Published:** 2025-09-01

**Authors:** Thu Tran, Navya Sankoorikkal, Lyca Intal, Thomas Lu, Saef Munir, Aman Jain, Wen Li, Antonio Corno, Amir Khan, Tina Findley

**Affiliations:** McGovern Medical School at UTHealth Houston; University of Texas-Houston Medical School; University of Texas-Houston Medical School; University of Texas-Houston Medical School; University of Texas-Houston Medical School; University of Texas-Houston Medical School; University of Texas-Houston Medical School; University of Texas-Houston Medical School; University of Texas-Houston Medical School; The University of Texas Health Science Center at Houston

**Keywords:** Hyperoxia, neurodevelopmental impairment, extremely premature neonates

## Abstract

Retrospective cohort study of extremely preterm infants (< 28 weeks’ gestational age and birth weight ≤ 1500 g) treated at a level IV academic hospital from January 2008 to July 2018 to assess if prolonged hyperoxia is an independent risk factor for severe neurodevelopmental impairment (NDI) or death.

Among 546 former extremely preterm infants, 327 (59.9%) were exposed to prolonged hyperoxia. Prolonged hyperoxia was associated with increased odds of severe NDI or death (OR 1.77, 95% CI 1.01 to 3.14) after adjusting for risk factors. When the components of the primary outcome were analyzed separately, prolonged hyperoxia was not associated with severe NDI among survivors (OR 1.39, 95% CI 0.74 to 2.63) but was associated with death (OR 3.20, 95% CI 1.23 to 9.59). In conclusion, prolonged exposure to oxygen is a significant and independent risk factor for development of long-term moderate to severe NDI including death.

## Introduction

Extremely premature neonates, born less than 28 weeks of gestation, face a high risk of respiratory distress syndrome due to surfactant deficiency(1). Lack of alveolar differentiation and surfactant synthesis by type II pneumocytes impair critical gas exchange, and many preterm neonates require mechanical ventilation after birth to meet oxygen demands(2, 3). Premature infants are particularly vulnerable to free oxygen radicals due to an immature antioxidant defense system. This leads to oxidative stress, resulting in DNA damage, systemic inflammation, impaired lung development, and alveolar hypoplasia – the underlying factors in the development of bronchopulmonary dysplasia (BPD) (4).

BPD affects approximately 43% of babies delivered before 29 weeks of gestation with mortality and long-term morbidities including neurodevelopmental impairment (NDI)(5–7). Delayed neurodevelopment is often attributed to prolonged intubation required for managing BPD. Few studies have investigated the effects of hyperoxia on neurodevelopmental outcomes in extremely premature neonates (5, 8, 9). We hypothesize that prolonged exposure to excessive oxygen concentration in the preterm infant is an independent risk factor for moderate to severe NDI.

## Methods

### PATIENT POPULATION

Eligible infants for this retrospective cohort study were extremely premature newborns (< 28 weeks’ gestational age and birth weight ≤ 1500 grams) admitted at Children’s Memorial Hermann Hospital, Houston, Texas, from January 2008 to July 2018. Study was approved by institutional review board with waiver of consent. Infants that were delivered at another facility and transferred to Children’s Memorial Hermann Hospital after one day of age or had early demise before three days of age were excluded due to limited and incomplete data. To minimize potential confounding in neurodevelopmental outcomes, infants with syndromic genetic conditions or other major congenital anomalies were also excluded from the study.

### EXPOSURE

Prolonged exposure to hyperoxia was defined as receiving fraction of inspired oxygen (FiO_2_) ≥ 0.4, delivered via non-invasive or invasive positive pressure ventilation, for a cumulative duration of at least three days. Positive pressure ventilation included high flow nasal cannula (HFNC), nasal continuous positive pressure airway pressure (nCPAP), or invasive mechanical ventilation. FiO_2_ administration was titrated by the bedside nurse to target peripheral oxygen saturation (SpO_2_) of 85–95% measured by continuous pulse oximetry during the study period.

### OUTCOME

Bayley Scales of Infant and Toddler Development exam was conducted at 22–26 months of corrected age to assess for NDI in the outpatient clinic by trained clinical psychologists. The second edition (Bayley II Mental Development Index) was used from 2008 to 2016 and the third edition (Bayley III) from 2016 to 2018 for NDI assessment. Moderate to severe NDI was defined as Bayley II Mental Developmental Index (MDI) below 70 or Bayley III cognitive score below 85, criteria that have demonstrated 97% concordance in prior studies(10, 11). For the purposes of this study, details on cerebral palsy, motor function, blindness, or deafness were not included in our assessment.

### DATA ANALYSIS

Continuous variables were summarized using means and standard deviations (SD). Categorical variables were summarized using frequencies and percentages. Two-tailed Student’s t test and chi-squared test were used to compare maternal and neonatal characteristics between hyperoxia and non-hyperoxia exposed groups. The primary outcome was severe NDI or death treated as a binary variable. With a sample size of 546 and a ratio of hyperoxia to non-hyperoxia of 1.5, we are powdered to detect an odds ratio (OR) as low as 1.6 with a power of 80% and an alpha level of 0.05, using a Chi-squared test. A common rule of thumb for sample size in multivariable logistic regression is to have at least ten observations in the least common outcome category for each variable included in the model. Thus, the sample size in the study was adequate for both the univariable and the multivariable analyses. Multivariable logistic regression analysis was used to determine if hyperoxia was an independent predictor of NDI or death after adjusting for baseline characteristics and co-morbidities including severe intraventricular hemorrhage (IVH) of grade 3 to 4, diagnosis of necrotizing enterocolitis (NEC), retinopathy of prematurity (ROP), and BPD defined using Jensen’s criteria(12). Jensen’s criteria assess neonates at 36 weeks’ postmenstrual age and classify the severity of BPD as follows: Grade 1 (mild) for those receiving ≤ 2 L/min of flow via nasal cannula, Grade 2 (moderate) for those requiring ≥ 2 L/min of flow via nasal cannula or non-invasive positive airway pressure, and Grade 3 (severe) for those requiring invasive mechanical ventilation(12). Those not on oxygen supplementation at 36 weeks’ postmenstrual age were classified as having no BPD(12). In addition, mean airway pressure (MAP) greater than 10 cmH_2_0 was used to adjust for severity of lung disease(13, 14). The same model was used to evaluate the relationship of hyperoxia and the individual components of the primary outcome. Statistical significance was defined as a p-value ≤ 0.05. All statistical analyses were performed in R Statistical Software (version 4.2.0; R Core Team 2022).

## Results

From January 2008 to July 2018, a total of 635 infants met the inclusion criteria of inborn extremely preterm infants that survived at least three days of life. Of these infants, 89 were excluded due to missing Bayley MDI assessments. A remaining total of 546 infants had Bayley assessments conducted at 22–26 months corrected age. Four infants in the non-hyperoxia exposed group were excluded in the logistic regression analysis due to missing method of oxygen delivery.

### PRIMARY OUTCOME

**Figure 1:**
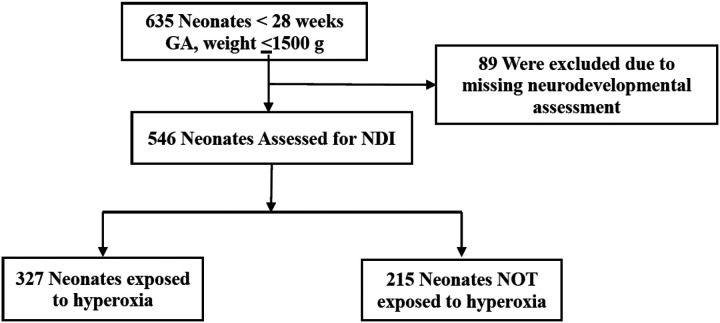
Study cohort and outcomes

Among the study cohort, 327 (59.8%) extremely preterm neonates were exposed to prolonged hyperoxia (Fig. 1). Prolonged exposure to hyperoxia in the extremely preterm population was a significant independent risk factor for moderate and severe NDI or death after adjusting for risk factors including gestational age and other co-morbidities (OR 1.77, 95% CI 1.01–3.14) (Tables 1 and 2). When the components of the primary outcome were analyzed separately, prolonged hyperoxia was not significantly associated with severe NDI among survivors (OR 1.39, 95% CI 0.74 to 2.63) (Table 2) but was significantly associated with death (OR 3.20, 95% CI 1.23 to 9.59).

### SECONDARY OUTCOME

In this cohort, IVH (OR 4.76, 95% CI 2.23–10.80), NEC (OR 2.96, 95% CI 1.48–5.88) and ROP (OR 2.26, 95% CI 1.20–4.25) was associated with increased odds of NDI or death when controlling for hyperoxia exposure and other baseline characteristics. Patients with severe BPD did not have increased odds of NDI (OR 1. 41, 95% CI 0.79–2.52) (Table 2). Gestational age, sex, and high MAP (> 10 cmH_2_0) were not significant independent risk factors for NDI or death (Table 2). However, utilizing subgroup analysis for survivors only, severe IVH (OR 3.75, 95% CI 1.58–9.16) and ROP (OR 2.63, 95% CI 1.32–5.22) remained significant risk factors for NDI or death (Table 3).

## Discussion

Our study aimed to investigate if prolonged hyperoxia exposure was independently associated with severe NDI or death. Our findings provide supporting evidence that infants requiring FiO_2_ ≥ 0.4 for cumulative duration of at least three days have significantly increased odds of NDI or death. Optimal oxygen saturation (SpO_2_) in preterm infants is still under debate. Three large clinical studies including Surfactant, Positive Pressure, and Oxygenation Randomized Trial (SUPPORT), Benefits of Oxygen Saturation Targeting (BOOST) II and Canadian Oxygen Trial (COT) investigated lower versus higher SpO_2_ target range in preterm infants less than 28 weeks’ gestational age. The SUPPORT trial found that lower SpO_2_ target range of 85–89% increased risk of death before discharge compared to SpO_2_ target range of 91–95%, but overall mortality rates or NDI at 18 to 22 months’ corrected age were no different(15)^–^(16). The BOOST II trial also demonstrated increased mortality rates in the lower SpO_2_ group after changing their methodology for technical oximeter-calibration during their trial(17). The COT trial found no difference in death or NDI at 18 months’ corrected age (18). A Cochrane review that included late preterm infants found oxygen administration and oxygen target range did not have significant effect on mortality, but liberal oxygen administration had a significant increased risk for combined adverse outcome of death or retrolental fibroplasia(19). In the Neonatal Oxygenation Prospective Meta-analysis (NeOProM) that included the above-mentioned studies, lower SpO2 target range of 85%−95% increased risk for death and NEC, but showed no difference in NDI(20).

A recent retrospective observational study of preterm neonates born less than 28 weeks’ gestational age found that white matter injury of the brain was more prevalent in neonates with lower targeted SpO_2_ (less than 90%) on magnetic resonance imaging (MRI), as well as in those with longer accumulated days requiring FiO_2_ greater than 0.21. These factors were also associated with poorer neurodevelopmental assessment scores(21). Supporting clinical studies, animal models have further demonstrated the detrimental impact of hyperoxia on brain injury, highlighting the potential mechanisms underlying neurological damage in premature neonates. During the infantile period, animals subjected to hyperoxia had brain injury due to various proposed mechanisms such as neuronal apoptosis, reduced brain mass, mitochondrial dysfunction, neurotrophin protection downregulation, reactive oxygen species (ROS) accumulation, inflammation-induced myelination disruption, as well as impairment of the axon-oligodendrocyte integrity and neuronal plasticity (21–29). *In vitro* MRI imaging and histochemical analysis of brain tissue in mice exposed to FiO_2_ > 85% for 14 days revealed reduced hippocampal and cerebellar volume as well as associated impaired spatial and recognition memory (17). Multiple animal studies have investigated the effects of prolonged hyperoxia on brain development, but there is a large variation in the animal model used as well as the degree of hyperoxia exposure (30–32).

Clinical studies have predominantly focused on BPD as a risk factor for poor neurodevelopmental outcomes. However, to the authors’ knowledge, none have examined prolonged cumulative exposure to FiO_2_ ≥ 0.40 as an independent risk factor (7, 33–35). There are several limitations to our study. First, this is a retrospective review and data on the corresponding SpO_2_ at the time of FiO2 administration was unavailable which could limit the direct correlation between patient saturation and supplemental oxygen titration. We acknowledge the lack of concurrent SpO_2_ data may cause difficulties in identifying critically ill patients that may need higher supplemental oxygen support. Our neonatal intensive care unit follows a protocol to keep SpO_2_ target range between 90–95% for premature infants. Additionally, SpO_2_ values alone do not capture the severity of lung disease. To address this limitation, MAP values were incorporated to account for the degree of lung severity and overall clinical illness. Another limitation is FiO_2_ levels is not a direct measure of O_2_ delivery to tissues. Although measured arterial oxygen levels would provide a more accurate assessment of whether FiO_2_ administration was excessive, the limited availability of arterial blood gas values constrained their use in this study. Lastly, in our patient population, there was a high mortality rate in the hyperoxia group. This introduces a potential bias, as death itself may have a more significant impact on outcomes than hyperoxia, potentially confounding the interpretation of the relationship between hyperoxia and clinical outcomes. Lastly, many infant deaths occurred in the hyperoxia group, and separating the mortality group from the hyperoxia group would have significantly reduced our sample size. As a result, both groups were included in our primary analysis.

## Conclusion

In conclusion, oxygen is a critical therapy in the management of extremely premature infants born under 28 weeks’ gestational age, but prolonged and excessive exposure to oxygen may be a significant and independent risk factor for development of long-term moderate to severe NDI including death. Our study adds to current literature by focusing on the administration of supplemental oxygen via positive pressure ventilation in preterm neonates and its association with death and adverse neurodevelopmental outcomes.

## Figures and Tables

**Table 1:** Baseline characteristics

Characteristics	No Hyperoxia (N = 215)	Hyperoxia (N = 327)	P-value
**Maternal**			
Maternal Age - yr	28.7 ± 7.1	29.2 ± 7.0	0.490
Race or ethnic group - no. (%)			
White/ Caucasian	43 (20.0)	82 (25.1)	0.170
North American Indian/ Native Alaskan)	0 (0)	1 (0.3)	0.417
Asian	4 (1.9)	5 (1.5)	0.768
Black/African American	102 (47.4)	133 (40.7)	0.120
Native Hawaiian/Other Pacific Islander	0 (0)	0 (0)	
Other	64 (29.8)	107 (32.)	0.469
Maternal hypertension - no. (%)	34 (16.1)	75 (23.1)	**0.048** *
Maternal diabetes - no. (%)	9 (4.3)	18 (5.6)	0.505
Intraamniotic Infection - no. (%)	57 (26.5)	80 (24.7)	0.635
Adequate antenatal glucocorticoid (≥2 doses - no. (%)	139 (65.3)	179 (55.4)	**0.023** *
Caesarean Section	141 (65.6)	231 (70.6)	0.214
Multiple birth - no. (%)	40 (18.7)	75 (23.4)	0.197
Prolonged rupture of membrane >18 hrs - no. (%)	63 (29.4)	104 (32.1)	0.514
**Neonatal**			
Gestational Age - wk	25.5 ± 0.9	24.7 ± 1.0	**<0.001** *
Male sex - no. (%)	161 (48.6)	123 (57.2)	0.050*
Mean birth weight (range) -- g	788 (403–1370)	668 (330–1115)	**<0.001** *
Mean APGAR at 5 mins (range)	6 (1–9)	5 (0–10)	**<0.001** *

† Four subjects had missing hyperoxia status.

* Denotes statistical significance

**Table 2:** Multivariable Regression Model for Neurodevelopmental Impairment (NDI)

Risk Factors/ Comorbidities	No NDI (N = 331)	NDI (N = 215)	Adjusted OR (95% CI)	*P*-value
**Hyperoxia Exposure**				
No Hyperoxia	156 (47.7%)	59 (27.4%)	Reference	--
Hyperoxia	171 (52.3%)	156 (72.6%)	1.77 (1.01–3.14)	**0.048** *
**Gestational Age (weeks)**				
≤ 24	41 (12.4%)	82 (38.1%)	Reference	--
24–25	102 (30.8%)	71 (33.0%)	0.94 (0.51–1.74)	0.84
25–26	104 (31.4%)	40 (18.6%)	0.54 (0.27–1.08)	0.08
26–27	84 (25.4%)	22 (10.2%)	0.55 (0.24–1.24)	0.16
**Gender**				
Female	170 (51.4%)	92 (42.8%)	Reference	--
Male	161 (48.6%)	123 (57.2%)	1.38 (0.87–2.18)	0.17
**Intraventricular Hemorrhage (IVH)**				
Not Severe (Grade 1–2)	318 (96.1%)	159 (74.0%)	Reference	--
Severe (Grade 3–4)	13 (3.9%)	56 (26.0%)	4.76 (2.23–10.80)	**<0.001** *
**Necrotizing Enterocolitis (NEC)**				
No NEC	312 (94.3%)	176 (81.9%)	Reference	--
NEC Present	19 (5.7%)	39 (18.1%)	2.96 (1.48–5.98)	**0.002** *
**Retinopathy of Prematurity (ROP)**				
No ROP	297 (91.4%)	117 (76.5%)	Reference	--
ROP Present	28 (8.6%)	36 (23.5%)	2.26 (1.20–4.25)	**0.011** *
**Bronchopulmonary Dysplasia (BPD)**				
None, Mild or Moderate	136 (45.8%)	30 (15.7%)	Reference	--
Severe	161 (54.2%)	161 (84.3%)	1.41 (0.79–2.52)	0.25
**Mean Airway Pressure (MAP)**				
**Hyperoxia Exposure**				
None or Low (<10 cm H_2_O)	306 (92.4%)	177 (83.5%)		
Medium or High (≥10 cm H_2_O)	25 (7.6%)	35 (16.5%)	1.50 (0.73–3.05)	0.26

* Denotes statistical significance ≤ 0.05. Reference group used for subgroup analysis indicated within table.

**Table 3:** Multivariable Regression Model for Neurodevelopmental Impairment (NDI) Among Survivors

Risk Factors/ Comorbidities	No NDI (N = 331)	NDI (N = 100)	Adjusted OR (95% CI)	*P*-value
**Hyperoxia Exposure**				
No Hyperoxia	156 (47.7%)	28 (28.0%)	Reference	--
Hyperoxia	171 (52.3%)	72 (72.0%)	1.39 (0.74–2.63)	0.31
**Gestational Age (weeks)**				
≤ 24	41 (12.4%)	26 (26.0%)	Reference	--
24–25	102 (30.8%)	34 (34.0%)	0.86 (0.43–1.76)	0.68
25–26	104 (31.4%)	28 (28.0%)	0.60 (0.28–1.30)	0.19
26–27	84 (25.4%)	12 (12.0%)	0.48 (0.18–1.23)	0.13
**Gender**				
Female	170 (51.4%)	37 (37.0%)	Reference	--
Male	161 (48.6%)	63 (63.0%)	1.38 (0.91–2.59)	0.11
**Intraventricular Hemorrhage (IVH)**				
Not Severe (Grade 1–2)	318 (96.1%)	84 (84.0%)	Reference	--
Severe (Grade 3–4)	13 (3.9%)	16 (16.0%)	3.75 (1.58–9.16)	**0.003** *
**Necrotizing Enterocolitis (NEC)**				
No NEC	312 (94.3%)	86 (86.0%)	Reference	--
NEC Present	19 (5.7%)	14 (14.0%)	1.77 (0.74–4.06)	0.19
**Retinopathy of Prematurity (ROP)**				
No ROP	297 (91.4%)	72 (73.5%)	Reference	--
ROP Present	28 (8.6%)	26 (26.5%)	2.63 (1.32–5.22)	**0.006** *
**Bronchopulmonary Dysplasia (BPD)**				
None, Mild or Moderate	136 (45.8%)	22 (23.7%)	Reference	--
Severe	161 (54.2%)	71 (76.3%)	1.28 (0.66–2.51)	0.47
**Mean Airway Pressure (MAP)**				
**Hyperoxia Exposure**				
None or Low (<10 cm H_2_O)	306 (92.4%)	83 (83.0%)		
Medium or High (≥ 10 cm H_2_O)	25 (7.6%)	17 (17.0%)	1.85 (0.84–4.01)	0.12

* Denotes statistical significance ≤ 0.05. Reference group used for subgroup analysis indicated within table.

## Data Availability

Data published in article can be made available via request.
